# Hyperuricemia and Its Association With the Severity and Complications of Congestive Heart Failure: A Systematic Review

**DOI:** 10.7759/cureus.45246

**Published:** 2023-09-14

**Authors:** Vijay P SN, Arturo P Jaramillo, Mohamed Yasir, Sally Hussein, Sanjana Singareddy, Nandhini Iyer, Tuheen Sankar Nath

**Affiliations:** 1 Internal Medicine, California Institute of Behavioral Neurosciences & Psychology, Fairfield, USA; 2 Research, California Institute of Behavioral Neurosciences & Psychology, Fairfield, USA

**Keywords:** hyperuricemia, congestive heart failure, cardiac complications, heart failure, elevated uric acid levels

## Abstract

Several studies have shown that an association exists between hyperuricemia and heart failure. Despite several innovative management strategies, heart failure is a significant cause of mortality worldwide. Hyperuricemia in heart failure patients leads to poorer outcomes. Additionally, hyperuricemia can be a strong surrogate marker for increased oxidative stress in heart failure patients. This oxidative stress leads to vascular endothelial damage and is linked to worsening heart failure and subsequent mortality. Hence, the measurement of serum uric acid levels in these patients can predict the present and future risk of complications of heart failure. Despite this knowledge, serum uric acid levels are not usually followed up in heart failure patients. This systematic review aims to give additional clarity to this association. We used research from the last twenty years (2002 to 2022) obtained from databases such as PubMed, PubMed Central (PMC), Google Scholar, and Science Direct. We used the Preferred Reporting Items for Systematic Review and Meta-analysis (PRISMA) 2020 guidelines. We removed duplicates, screened articles on the basis of title and abstract, applied eligibility criteria, and performed quality appraisal. Eventually, 15 articles were selected for review. There were 12 observational studies, two randomized controlled trials, and one meta-analysis. Our review showed that serum uric acid elevation is associated with the severity and complications of congestive heart failure. Serum uric acid can serve as a useful surrogate marker of oxidative stress in congestive heart failure (CHF) patients. The role of xanthine oxidase inhibitors needs to be evaluated further in CHF patients.

## Introduction and background

Heart failure affects over 26 million individuals worldwide and, due to the high levels of associated morbidity and resource consumption, places a significant burden on healthcare systems [1, 2]. Hospitalization is an important indicator of the stress on congestive heart failure (CHF) patients and healthcare infrastructure. In the United States, more than a million hospital admissions and readmissions occur due to heart failure annually [3]. Heart failure-related 30-day mortality and readmission risk have not significantly decreased over time [4, 5]. Thus, if health systems are to improve outcomes for patients with heart failure, new strategies to enhance clinical decision-making and care are required.

Heart failure is a condition that worsens over time and can significantly impact a person's quality of life and increase their risk of death [6, 7]. It is characterized by elevated ventricular filling pressures and poor organ perfusion, which can lead to dysregulated homeostasis and metabolic issues. One common metabolic derangement in heart failure patients is hyperuricemia, which is characterized by high levels of uric acid in the blood. Uric acid is a by-product of purine breakdown, which is catalyzed by the enzyme xanthine oxidase (XO) and can produce harmful oxygen radicals [8]. 

In CHF, hyperuricemia is associated with severe symptoms and an increased risk of complications, including mortality [9-12]. It has been found that hyperuricemia is closely related to atrial fibrillation, which can worsen heart failure [13]. It has been found that there is an increased XO activity in CHF patients. This leads to free radical generation, which causes vascular endothelial damage and increased cardiac afterload. Hence, hyperuricemia, which is a product of xanthine oxidase activity, is seen in such patients. Noxious stimuli such as some cytokines and the sustained activation of the renin-angiotensin system in CHF patients can lead to increased xanthine oxidase activity [14].

Despite this knowledge, it is not yet clear whether hyperuricemia is a direct cause of heart failure progression or simply a marker of a worse prognosis. Several studies have been published on the significance of serum uric acid levels in heart failure patients. This has led us to conduct a thorough review of these studies to gain more insight into the matter. Our objective is to determine the potential link between hyperuricemia and the severity of congestive heart failure, as well as its complications, such as mortality.

## Review

Methods

This review utilized the Preferred Reporting Items for Systematic Review and Meta-Analyses (PRISMA) 2020 guidelines [15].

Search Strategy and Data Collection

A systematic search for articles published between 2002 and 2022 was carried out on PubMed, PubMed Central (PMC), Google Scholar, and Science Direct. Database search was done utilizing the three keywords: 'hyperuricemia', 'uric acid', and 'congestive heart failure'. In Google Scholar and Science Direct, these keywords were first used separately and subsequently combined using Boolean "AND". In PubMed and PMC, keywords and synonyms of keywords were combined using Boolean "OR". The two separate concepts were combined using the Boolean "AND", to create a Medical Subject Heading (MeSH) search strategy. The final MeSH keyword entered was as follows:

"Hyperuricemia OR asymptomatic hyperuricemia OR elevated uric acid levels OR high uric acid levels OR ("hyperuricemia/complications"[Majr] OR "hyperuricemia/mortality"[Majr] OR "hyperuricemia/physiopathology"[Majr]) AND congestive heart failure OR congestive cardiac failure OR reduced ejection fraction OR systolic heart failure OR ("heart failure/complications" [Majr] OR "heart failure/mortality" [Majr] OR "heart failure/physiopathology" [Majr])". Two reviewers collected data independently. Table 1 highlights the databases searched along with the search strategy which was employed to obtain relevant results.

**Table 1:** Databases searched and the search strategy used

Database	Keywords	Search Strategy	Filters used	No. of records
PubMed Advanced Search	Hyperuricemia, asymptomatic hyperuricemia, elevated uric acid levels, high uric acid levels, congestive heart failure, congestive cardiac failure, reduced ejection fraction, systolic heart failure	Hyperuricemia OR asymptomatic hyperuricemia OR elevated uric acid levels OR high uric acid levels OR ("hyperuricemia/complications"[Mesh] OR "hyperuricemia/mortality"[Mesh] OR "hyperuricemia/physiopathology"[Mesh] ) AND congestive heart failure OR congestive cardiac failure OR reduced ejection fraction OR Systolic heart failure OR ( "heart failure/complications"[Mesh] OR "heart failure/mortality"[Mesh] OR "heart failure/physiopathology"[Mesh] )	Free full text, books and documents, clinical trials, meta-analyses, randomized controlled trials, reviews, systematic reviews, between 2002 to 2022, humans, adults 19+ years, English language, exclude preprints	180
Google Scholar Advanced Search	Hyperuricemia, uric acid, congestive heart failure	All in title: hyperuricemia OR uric acid AND congestive heart failure	Published in the last 20 years	11
Science Direct	Hyperuricemia, uric acid, congestive heart failure	All in title: hyperuricemia OR uric acid AND congestive heart failure	Published in the last 20 years	698

Eligibility Criteria and Study Selection

Endnote (Clarivate, Philadelphia, United States) was used to detect and eliminate duplicates. The collected publications were then screened on the basis of title and abstract. Completely irrelevant articles were removed. After this process, they were assessed for eligibility based on inclusion and exclusion criteria. The articles published between 2002 and 2022 in the English language, with studies conducted on human subjects, involving only the adult population, and containing the full text were considered. All research that did not fall between 2002 and 2022, not published in English, unpublished grey literature, animal studies, and studies involving children were excluded. The studies which were eligible were then subjected to quality assessment.

Quality Assessment

The Modified Newcastle-Ottawa Scale was used to quality-check the observational studies. For clinical trials, the Cochrane Risk of Bias tool was used, and for meta-analysis, the assessment of multiple systematic reviews (AMSTAR) checklist was used. The studies with a minimum score of 70% and above were considered to be of high quality. Two authors conducted a quality assessment, and a third reviewer was invited to settle any disputes that may arise during the discussion. The quality appraisal tools used in this study are depicted in Table 2.

**Table 2:** Quality appraisal tools used in this study AMSTAR - assessment of multiple systematic reviews

Type of study	Quality appraisal tool	Number of studies
Observational studies	Modified Newcastle-OttawaScale	12
Randomized controlled trials	Cochrane Risk of Bias tool	2
Meta-analysis	AMSTAR checklist	1

**Figure 1:**
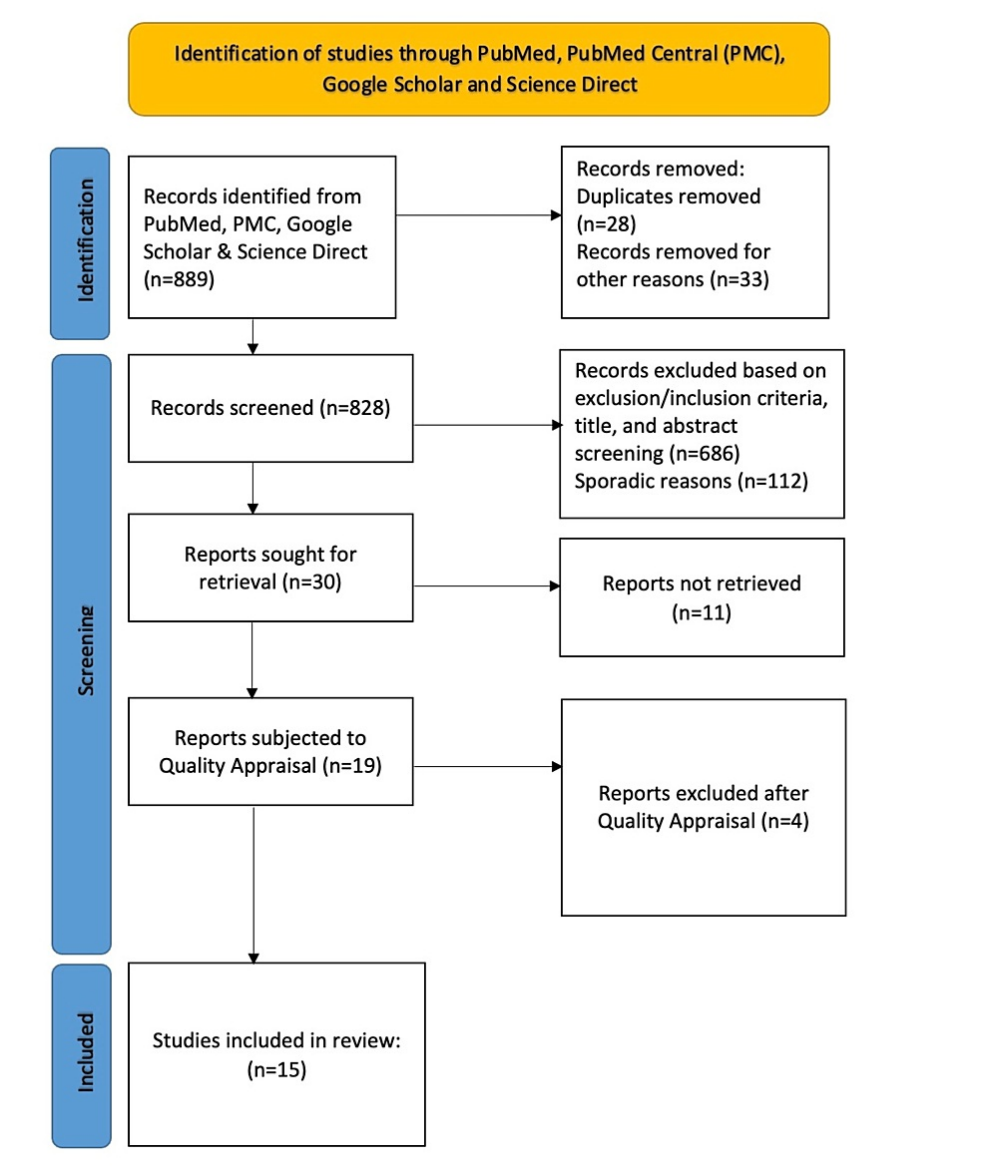
Preferred Reporting Items for Systematic Reviews and Meta-Analyses (PRISMA) 2020 flowchart used for this review n: Number of records

The PRISMA flow diagram depicting the screening procedure is shown in Figure 1.

Results

A systematic search of various databases like PubMed, PubMed Central, Google Scholar, and Science Direct yielded a total of 889 articles. To remove duplicates, the EndNote tool (Clarivate, Philadelphia, USA) was used, resulting in the removal of 28 duplicates. Additionally, 33 articles were removed as they were not relevant. After careful screening of titles, abstracts, and eligibility criteria, 686 articles were removed, leaving the remaining relevant articles for further analysis.

We removed an additional 112 articles for sporadic reasons, and eventually, 30 articles were selected for full-text review. Out of these, the full text of 11 articles could not be retrieved. Nineteen articles were successfully retrieved and were subject to a quality appraisal. Four articles were eliminated because they did not obtain the minimum score of 70% set during quality appraisal. A total of 15 articles were selected for the final review. This included two clinical trials, 12 observational studies, and one meta-analysis, which were found to be of high quality during the quality appraisal check using the Cochrane Risk of Bias Tool, the Modified Newcastle-Ottawa Scale, and the AMSTAR checklist. The Modified Newcastle-Ottawa Scale used for quality appraisal of observational studies is shown in Table 3.

**Table 3:** Modified Newcastle-Ottawa Scale used for quality appraisal of observational studies Yes - one point given; ! - uncertain (no points given)

Newcastle-Ottawa Criteria	Anker et al. 2003 [16]	Krishnan 2009 [17]	Ogino et al. 2010 [18]	Eisen et al. 2013 [19]	Krishnan et al. 2012 [20]	Deis et al. 2022 [21]	Pascual-Figal et al. 2007 [22]	Gu et al. 2018 [23]	Wannamethee et al. 2018 [24]	Ekundayo et al. 2010 [25]	Okazaki et al. 2016 [26]	Joho et al. 2019 [27]
Representativeness of the exposed cohort	Yes	Yes	Yes	Yes	Yes	Yes	Yes	Yes	Yes	Yes	Yes	Yes
Selection of external control	!	!	Yes	!	Yes	!	!	!	Yes	Yes	!	!
Ascertainment of exposure	Yes	Yes	Yes	Yes	Yes	Yes	Yes	Yes	Yes	Yes	Yes	Yes
Outcome of interest not present at the start of study	Yes	Yes	Yes	Yes	Yes	Yes	Yes	Yes	Yes	Yes	Yes	Yes
Comparability of main factor	Yes	Yes	Yes	Yes	Yes	Yes	Yes	Yes	Yes	Yes	Yes	Yes
Comparability of additional factor	!	!	!	!	!	!	!	!	!	!	!	!
Assessment of outcome	Yes	Yes	Yes	Yes	Yes	Yes	Yes	Yes	Yes	Yes	Yes	Yes
Sufficient follow-up time	Yes	Yes	Yes	Yes	Yes	Yes	Yes	Yes	Yes	Yes	Yes	Yes
Adequacy of follow-up	Yes	Yes	Yes	Yes	Yes	Yes	Yes	Yes	Yes	Yes	Yes	Yes
Total score	7/9	7/9	8/9	7/9	8/9	7/9	7/9	7/9	8/9	8/9	7/9	7/9

Study Characteristics

After a thorough selection analysis of 15 published articles, a total of 22,131 patients were identified. The table below summarizes the outcome of each article and provides the ultimate consensus on the association between hyperuricemia and the severity and complications of congestive heart failure. The summary of features and outcomes of individual studies included in this review are depicted in Table 4.

**Table 4:** Summary of the characteristics and outcomes of individual studies included in this systematic review CHF - congestive heart failure; CAD - coronary artery disease; HFpEF - heart failure with preserved ejection fraction; MACE - major adverse cardiovascular events; AHF - acute heart failure; ROS - reactive oxygen species; XO - xanthine oxidase; HFrEF - heart failure with reduced ejection fraction; LVEF - left ventricular ejection fraction

Author and year of publication	Study type	Population under study	Intervention if any	Sample size	Follow-up duration	Conclusion
Anker et al. 2003 [16]	Cohort study	Adults		294	12 months	Hyperuricemia is a strong and independent marker of poor prognosis in moderate to severe CHF patients.
Krishnan 2009 [17]	Cohort study	Adults		4912	29 years	In young general community dwellers, hyperuricemia is an independent risk factor for heart failure.
Ogino et al. 2010 [18]	Case-control study	Adults		98		Upregulated XO activity is involved in impairment in CHF. Uric acid lowering without XO inhibition may not influence the hemodynamic impairment seen in CHF.
Eisen et al. 2013 [19]	Cohort study	Adults		2939	8 years	In stable CAD patients, high uric acid levels are associated with a future risk of heart failure, but this risk is diminished after adjusting for traditional CAD risk factors.
Krishnan et al. 2012 [20]	Cohort study	Adults		2269	24 years	Hyperuricemia in young adults is a marker for subsequent heart failure.
Deis et al. 2022 [21]	Cohort study	Adults		228	9 years	In advanced heart failure patients, an association between central hemodynamics and uric acid levels exists but it is unknown if this relationship is causal.
Pascual-Figal et al. 2007 [22]	Cohort study	Adults		212	20 months	Elevated uric acid levels are a long-term prognostic marker for death and/or new CHF hospital readmission in hospitalized patients with acute heart failure and systolic dysfunction.
Gu et al. 2018 [23]	Cohort study	Adults		1009	7 years	In hypertensive patients, hyperuricemia is associated with the incidence of new-onset HFpEF and MACEs.
Wannamethee et al. 2018 [24]	Cohort study	Adults		3440	15 years	In old hypertensive men on treatment, hyperuricemia is associated with an increased risk of heart failure.
Ekundayo et al. 2010 [25]	Cohort study	Adults		5461	8 years	Among a group of older adults living in the community, hyperuricemia when it is due to an increased XO activity is associated with an increased heart failure risk.
Okazaki et al. 2016 [26]	Cohort study	Adults		889	6 months	In acute heart failure patients undergoing emergent hospitalization, hyperuricemia on admission is associated with adverse outcomes.
Joho et al. 2019 [27]	Cohort study	Adults		139	4.5 years	In heart failure patients with sympathetic overactivation, hyperuricemia may have a deleterious effect on prognosis and cardiac function.
Yokota et al. 2018 [28]	Randomized controlled trial	Adults	Febuxostat vs, placebo	200	6 months	In chronic heart failure patients with hyperuricemia, febuxostat might improve clinical outcomes through reduced ROS emission originating from decreased XO activity rather than lowered serum uric acid levels.
Givertz et al. 2015 [29]	Randomized controlled trial	Adults	Allopurinol vs. placebo	253	6 months	In high-risk patients with HFrEF and hyperuricemia, XO inhibition with allopurinol failed to improve exercise capacity, clinical status, quality of life, and LVEF at 24 weeks.
Huang et al. 2019 [30]	Meta-Analysis	Adults		12359		In acute heart failure patients, elevated serum uric acid levels are an independent predictor of all-cause mortality and the combined endpoint of death or hospital readmission. Measurement of serum uric acid may help in the risk stratification of adverse outcomes in such patients.

Discussion

Several studies have demonstrated that heart failure is frequently associated with hyperuricemia. The importance of these associations, however, continues to be controversial. There is also a large volume of published literature that suggests that the relationship between hyperuricemia and congestive heart failure should be seriously considered.

Pathophysiological Link of Uric Acid With Heart Failure

In a study by Anker et al., hyperuricemia was suggested to reflect raised xanthine oxidase activity in congestive heart failure. Oxygen free radicals are generated by the xanthine oxidase system and the generated free radicals provide the pathophysiological link of uric acid with a variety of deleterious effects, including increased cytokine production, apoptosis, and endothelial dysfunction. They suggested that the effects of xanthine oxidase, which was expressed only in the myocardium, were local and not systemic [16].

In a study by Krishnan, it was suggested that hyperuricemia could be a marker of abnormal oxidative metabolism. The level of serum uric acid in the human body is an indicator of oxidative stress. It has been found that hyperuricemia-induced reduction of nitric oxide production leads to endothelial dysfunction. There may also be a connection between hyperuricemia and heart failure through inflammation, as asymptomatic hyperuricemia is associated with higher levels of serum markers of inflammation, such as C-reactive protein, interleukin-6, and neutrophil count. It has been found that in patients with heart failure who have hyperuricemia, higher levels of markers indicating endothelial activation, such as the soluble intercellular adhesion molecule-1, as well as inflammatory markers like interleukin-6, tumor necrosis factor, and its receptors are seen [17].

In another study by Ogino et al., it was suggested that xanthine oxidase, an enzyme in human physiology, is a significant cause of the accumulation of reactive oxygen species. These reactive oxygen species, derived from xanthine oxidase, could contribute to a variety of harmful processes within congestive heart failure pathophysiology, such as endothelial dysfunction, inflammatory activation, and metabolic impairment [18].

Eisen et al. suggested that high uric acid levels may be a marker of oxidative stress and can lead to endothelial damage by reducing nitric oxide production. This could potentially worsen heart failure. Additionally, hyperuricemia has been linked to increased levels of inflammatory markers like C-reactive protein, interleukin-6, and neutrophil count, which may also be related to heart failure [19].

Serum Uric Acid as a Biomarker in Congestive Heart Failure

In a study by Krishnan et al., it was found that increased levels of serum uric were an independent predictor of the markers of inflammation such as intercellular adhesion molecule 1, tumor necrosis factor-alpha, soluble tumor necrosis factor receptor (sTNFR)1, sTNFR2, and interleukin 6. They also suggested that using elevated serum uric acid levels as a marker for the development of subclinical heart failure in young adults may allow for preventive measures [20]. This study had a potential for volunteer bias and a relative lack of ethnic minorities in the study population. Being a large epidemiologic study, residual confounding by unmeasured confounders could not be ruled out.

In a study by Pascual-Figal et al., it was found that hyperuricemia in patients hospitalized with acute heart failure and left ventricular systolic dysfunction was associated with a higher risk of mortality and future hospitalizations. This led the authors to suggest the inclusion of routine measurement of serum uric acid levels in risk models in acute heart failure patients with a hospitalization history [22]. This study was limited by the fact that uric acid levels were not measured during follow-up visits of these patients. Hence, it was not possible to determine if uric acid levels improved with symptomatic improvement of heart failure. Also, B-type natriuretic peptide (BNP) levels, which are strongly linked to the prognosis and severity of CHF, were not measured in this study.

Huang et al., in their 2019 meta-analysis, found that elevated serum uric acid levels were independent predictors of all-cause mortality and the combined endpoint of death or hospital readmission in acute heart failure patients. They concluded that measurement of serum uric acid levels may help in risk stratification of acute heart failure patients [30]. This study had a small sample size and a short follow-up period, which may have affected the results. In addition, it suffers from a lack of generalizability as the majority of the people analyzed were elderly.

Complications of Hyperuricemia in Hypertensive Patients and Patients With Acute or Chronic Heart Failure

In their 2018 study, Gu et al. found that hyperuricemia could predict the onset of heart failure with preserved ejection fraction (HFpEF) and major adverse cardiovascular events (MACEs). This was true even when other risk factors were present in hypertensive patients with left ventricular hypertrophy and suspected left ventricular diastolic dysfunction. They also suggested that arterial stiffness could increase with increasing serum uric acid levels, and this, in turn, could potentially cause or exacerbate HFpEF [23]. This study was a retrospective cohort study and as a result, there is the possibility of confounding and selection bias. In addition, the study cohort was from a single institution and this potentially limits the external validity.

Wannamethee et al., in their study, suggested that in patients with essential hypertension, hyperuricemia could be a compensatory response to counteract excessive oxidative stress and thus represent a marker of increased xanthine oxidase activity. They concluded that serum uric acid has an association with an increased risk of heart failure in older men on treatment with antihypertensives. They also suggested that since xanthine oxidase activity is difficult to measure, serum uric acid can be used as a surrogate marker of oxidative stress [24]. This study also has a lack of generalizability as the population that was studied was predominantly older white hypertensive men. Moreover, serum uric acid was measured only once; hence, it is not possible to comment on the trends of serum uric acid with symptomatic improvement or worsening. 

Deis et al., in their study, found that elevated serum uric acid levels were a strong and independent predictor of all-cause mortality in addition to the need for placement of a left ventricular assist device and heart transplant. There was a 15% increased risk of all-cause mortality with every 10% increase in plasma uric acid levels. This study also found a strong association between uric acid levels, left-sided filling pressures, and central hemodynamics [21]. A cause-effect relationship could not be established because of the retrospective nature of the study. There is also the possibility of selection bias as the patients recruited for this study were referred for right heart catheterization to a single specialized center.

 In another study by Okazaki et al., the authors concluded that hyperuricemia in emergently hospitalized acute heart failure patients was associated with worse outcomes. They hypothesized that the profound hypoxemia in acute heart failure patients compared to chronic heart failure patients leads to increased activation of the enzyme xanthine oxidase and, thereby, uric acid, which is the end product of this enzyme activity. They also found that high uric acid levels were associated with increased all-cause mortality and heart failure events [26]. The study population included only acute heart failure patients admitted to the intensive care unit (ICU); hence, there is the possibility that this study may not be representative of patients who had milder forms of acute heart failure and were admitted to the general wards. There is also the possibility of unmeasured confounders affecting the results as this study was not a prospective randomized controlled trial. 

A meta-analysis conducted by Huang et al. revealed that high uric acid levels were independent predictors of all-cause mortality and the combined endpoint of death or readmission in acute heart failure (AHF) patients. In hyperuricemic AHF patients, there is a 43% increased risk of all-cause mortality and a 68% increased risk of death or hospital readmission. A 1mg/dl increase in serum uric acid levels increases the risk of death due to all causes by 11% and the risk of death or hospital readmission by 12% in AHF patients. They also found that in acute decompensated heart failure, uric acid level at the time of admission was a strong and independent predictor of all-cause mortality and hospital readmission 30 days after discharge. Hyperuricemic patients with heart failure with preserved ejection fraction had poorer outcomes compared to those with heart failure with reduced ejection fraction [30]. The small sample size, inherent limitations of original studies, brief follow-up period, and studies involving predominantly elderly people limiting generalizability are the major drawbacks of this study.

Benefits of Decreasing Serum Uric Acid or Xanthine Oxidase Activity in CHF Patients

Deis et al., in their study, opined that there was no significant benefit of lowering serum uric acid through xanthine oxidase inhibition either with allopurinol or oxypurinol in CHF patients. They also suggested that direct uric acid lowering by uricosuric agents without xanthine oxidase inhibition did not have any significant benefit in CHF patients [21]. Additional prospective double-blind multicenter randomized controlled trials with large sample sizes and adequate follow-up duration are needed before any conclusions can be made on the cardiovascular benefits of pharmacological uric acid-lowering therapies. 

Ekundayo et al., in their analysis, suggested that it is important to determine the pathophysiology of hyperuricemia in any given patient with congestive heart failure and establish whether hyperuricemia is due to renal dysfunction and diuretic use or increased xanthine oxidase activity. They also suggested that hyperuricemia may be a predictor of heart failure when it occurs due to increased xanthine oxidase activity and that therapies inhibiting xanthine oxidase activity in such congestive heart failure patients may be beneficial [25]. Another study found that treatment with the uricosuric agent benzbromarone in CHF patients had no effect on BNP levels or on left ventricular functional parameters. According to the authors, treating high levels of uric acid without inhibiting the xanthine oxidase pathway through uricosuric therapy does not have an impact on hemodynamic variables in the pathophysiology of CHF. Therefore, the severity of congestive heart failure was due to increased xanthine oxidase activity and not hyperuricemia [18].

Joho et al. studied the interplay between hyperuricemia and hyperadrenergic state in heart failure patients. They suggested that sympathetic nerve activity or norepinephrine activates uric acid and its precursors, such as hypoxanthine and xanthine. They also suggested that hyperuricemia is a potent surrogate marker of increased levels of xanthine oxidase. They postulated that increased xanthine oxidase activity produced reactive oxygen species, which caused sympathetic overactivation, and this, in turn, caused beta-adrenergic receptor-induced cardiomyocyte apoptosis and contributed to poor prognosis in heart failure patients. Sympathetic overactivity can also directly produce reactive oxygen species, and hence, both xanthine oxidase and sympathetic overactivity may be interrelated in the pathophysiology of CHF. They concluded that hyperuricemia in a CHF patient in a hyperadrenergic state may be an ominous predictor of worse outcomes [27]. Thus, beta blockers are indispensable in the treatment of heart failure, and future trials need to be carried out to establish the efficacy of xanthine oxidase inhibitors in the treatment of congestive heart failure. 

The Xanthine Oxidase Inhibition for Hyperuricemic Heart Failure Patients (EXACT-HF) trial hypothesized that a reduction in xanthine oxidase levels in CHF patients will lead to clinical improvement and a fall in BNP levels at 24 weeks of follow-up. After 24 weeks, it was found that no significant benefit was found in lowering uric acid levels through xanthine oxidase inhibition in CHF patients [29]. This study was limited by a small sample size and brief follow-up duration. 

A meta-analysis conducted by Huang et al. revealed that despite xanthine oxidase inhibitors significantly reducing uric acid levels, they did not have a significant impact on improving outcomes in some clinical trials. However, after analyzing these trials post hoc, it was found that patients with higher serum uric acid levels experienced benefits that correlated with the degree of reduction in serum uric acid. They also suggested that there was a 2.1-fold higher risk of all-cause mortality in hyperuricemic acute or chronic heart failure patients [30]. This study had issues with generalizability as most of the study population were elderly patients, and a subgroup analysis was not conducted.

Limitations

This study is not without limitations. Even though several observational studies have established an association between high serum uric acid levels and the severity and complications of CHF, there was a lack of a uniform cut-off value for hyperuricemia in most of the studies. Many studies used an arbitrary value between 6 mg/dl and 7mg/dl to define hyperuricemia. Many patients with severe symptoms of congestive heart failure need to be on diuretics. This important variable was not adequately addressed in most of the individual studies. Increased xanthine oxidase activity was hypothesized to be the reason for uric acid elevation in CHF patients, but xanthine oxidase activity was not directly assayed in many of the studies. 

## Conclusions

Heart failure remains a significant cause of illness and death worldwide despite notable improvements in treatment options. It has been known for long that hyperuricemia is associated with heart failure. In this context, serum uric acid testing can be a simple yet effective marker of increased oxidative stress in the milieu of CHF and can predict further deterioration. It can also be used in the prognosis of such patients. In our systematic review, we found that all twelve observational studies and one meta-analysis included in the review support the theory that an association exists. One clinical trial suggested that there is no benefit in lowering uric acid levels through xanthine oxidase inhibition but this study was limited by a small sample size and a short follow-up period. Future studies are needed with uniformly defined cut-off values for hyperuricemia with subgroup analysis of the type, severity, and onset of heart failure with hyperuricemia. Studies have also shown that direct uric acid lowering using uricosuric agents has no benefit and that xanthine oxidase activity is the main pathophysiological link to heart failure. Since it is difficult to measure xanthine oxidase activity directly, serum uric acid can be a useful surrogate marker. Before deciding on the role of xanthine oxidase inhibitors in CHF, more prospective double-blind randomized placebo-controlled trials need to be conducted in a large population with adequate follow-up duration.
